# Personalized environmental risk communication and cancer screening intent among rural private well users

**DOI:** 10.1016/j.pmedr.2026.103620

**Published:** 2026-09-02

**Authors:** Victoria Clower, Andrew Hansen, Jeff Jones, Asli Aslan

**Affiliations:** a Institute for Water and Health, Georgia Southern University, Savannah, GA 31419, USA; b Department of Health Policy and Community Health, Jiann-Ping Hsu College of Public Health, Georgia Southern University, Statesboro, GA 30460, USA; c Department of Biostatistics, Epidemiology, and Environmental Health Sciences, Jiann-Ping Hsu College of Public Health, Georgia Southern University, Statesboro, GA 30460, USA

**Keywords:** Cancer screening, Environmental risk communication, Health belief model, Private wells, Rural health, Social determinants of health

## Abstract

**Objective:**

Private well contamination poses underappreciated cancer risks in rural communities where screening rates remain low. We examined whether personalized well water testing and environmental health education framed by the Champion Health Belief Model (CHBM), influence cancer screening intent among rural private well owners in southeast Georgia.

**Methods:**

Between July and October 2025, a 14-week study enrolled 100 private well owners assigned to an education-plus-testing group (*n* = 50) or a testing-only group (*n* = 50). Pre- and post-intervention surveys assessed CHBM constructs and screening intent. Binary logistic regression identified psychosocial and environmental predictors.

**Results:**

Overall, 68% of wells contained at least one contaminant, with radon exceeding 300 pCi/L in 56% of samples. Cancer screening intent rose 32% in the education-plus-testing group and 36% in the testing-only group. Understanding personalized well results was the strongest predictor of screening intent (Adjusted Odds Ratio (AOR) = 4.75, *p* = .029), and detection of any contaminant was independently associated with higher intent (AOR = 2.67, *p* = .039).

**Conclusions:**

Personalized environmental exposure information may serve as a social-determinants-informed cue to action, bridging environmental risk communication and cancer prevention in underserved rural communities.

## Highlights

• Personalized well testing was associated with greater cancer screening intent.
• Understanding well results were strongly associated with cancer screening intent
• Over half of rural private wells contained at least one cancer-associated contaminant.
• Radon frequently exceeded the study's action threshold in private well water.
• Environmental risk communication was associated with higher screening intent.

## 1 Introduction

Rural communities in the United States face environmental exposures and reduced access to cancer prevention services. Approximately 15% of Americans rely on unregulated private groundwater wells not monitored under the Safe Drinking Water Act, concentrating environmental health risks among rural and socially disadvantaged households (Environmental Protection Agency (EPA), 2024). Private well water may contain arsenic, radon, nitrate, and lead; however, evidence regarding carcinogenicity, exposure pathways, and health risks differs across contaminants (International Agency for Research on Cancer (IARC), 2024; MacRae et al., 2024).

Social determinants of health, including geography, socioeconomic status, and access to preventive services, shape environmental exposure risk and cancer outcomes. Rural Georgia counties experience disproportionately high mortality from lung, colorectal, and prostate cancers, with southeast Georgia identified as a region of concentrated cancer burden (Moore et al., 2022). Cancer screening rates compound this inequity: only 5% of high-risk individuals complete recommended lung cancer screening, and colorectal screening rates in southeast Georgia are as low as 33% (Georgia CORE, 2024). Cost, transportation, limited healthcare infrastructure, and reduced health literacy contribute to delayed diagnoses and poorer survival (Lee et al., 2023; Brock et al., 2024). Aruma et al. (2024) found medical mistrust was the strongest predictor of reduced cancer screening uptake among rural women after controlling for travel distance, cancer fatalism, and rurality. Thus, improving access alone may be insufficient; interventions must also address factors shaping screening decisions. Personalized environmental risk information may make health risks locally and individually meaningful.

Risk perception may link environmental awareness to preventive behavior. Individuals are less likely to act on invisible, probabilistic, or temporally distant threats, a profile that fits chronic groundwater contaminant exposure (Ntaoti, 2019). Personalized, actionable risk information may shift risk perceptions and motivate protective behavior. MacDonald Gibson et al. (2022) found targeted risk communication increased private well testing engagement, and Schultz et al. (2025) reported tailored outreach motivated 13% of participants to test their wells within one year. Individually meaningful environmental feedback may therefore function as a “cue to action,” a central Health Belief Model construct.

The Champion Health Belief Model (CHBM) provides a framework for understanding cancer screening decisions (Champion and Skinner, 2008). CHBM posits that preventive action is more likely when individuals perceive susceptibility to a serious threat, believe screening will reduce risk, perceive manageable barriers, and feel confident in their ability to act. The model has been applied to breast and colorectal cancer screening (Lau et al., 2020; Masoudiyekta et al., 2018) but not to environmental exposure contexts. Private well testing provides individualized contaminant results that may activate CHBM constructs and motivate screening behavior.

This study examines whether environmental health education and free private well testing, grounded in the CHBM, influence cancer screening intent among rural private well owners in southeast Georgia. We assess pre- and post-intervention changes in CHBM constructs and whether understanding of personalized water quality results predicts screening intent. This research connects environmental risk communication with cancer prevention and extends the CHBM to a rural environmental health context.

## 2 Methods

### 2.1 Study area

This study was conducted in Tattnall, Bulloch, and Evans counties in southeast Georgia, predominantly rural counties characterized by socioeconomic disadvantage and elevated cancer mortality. Median household incomes range from $42,000 to $51,868, with poverty rates exceeding state and national averages (Rural Health Information Hub (RHIhub), 2024). Approximately 1.7 million Georgians rely on private wells for drinking water; in the study counties, many households depend on unregulated groundwater wells, leaving residents responsible for testing and maintenance (Georgia Rural Health Innovation Center (GRHIC), 2019). Southeast Georgia has been identified as a cancer mortality hot spot for colorectal, prostate, and lung cancers, and cancer accounts for one in five deaths statewide (Moore et al., 2022; Department of Public Health (DPH), 2019).

### 2.2 Participants and interventions

The study was conducted over a 14-week period between July and October 2025, during which pre- and post-intervention surveys, educational activities, and private well water sampling were completed. Participants were recruited via printed flyers at local businesses, churches, and community centers; online advertisements in regional social media groups; and word-of-mouth referrals through community leaders. Eligibility required participants to be at least 18 years of age, reside within a study county, and use a private well as their primary drinking water source for at least one year. Eligibility was assessed as part of the study consent and pre-intervention survey, and all individuals who completed the eligibility screening met the inclusion criteria. Participants were enrolled sequentially into two intervention groups rather than randomly assigned. For Group 1, community members were invited to monthly church-based community dinners where they had the opportunity to participate in an environmental health education and listening session and enroll in the study. Interested participants completed informed consent and the pre-intervention survey on site, participated in the educational session, and completed an immediate post-education survey. Participants were subsequently contacted to schedule free private well water testing. Well water samples were collected on Mondays and Tuesdays of each week, with up to 5 samples collected per day, and were submitted to a third-party laboratory for analysis. After receiving their individual laboratory results by email, participants completed a final post-testing survey.

**Fig. 1:**
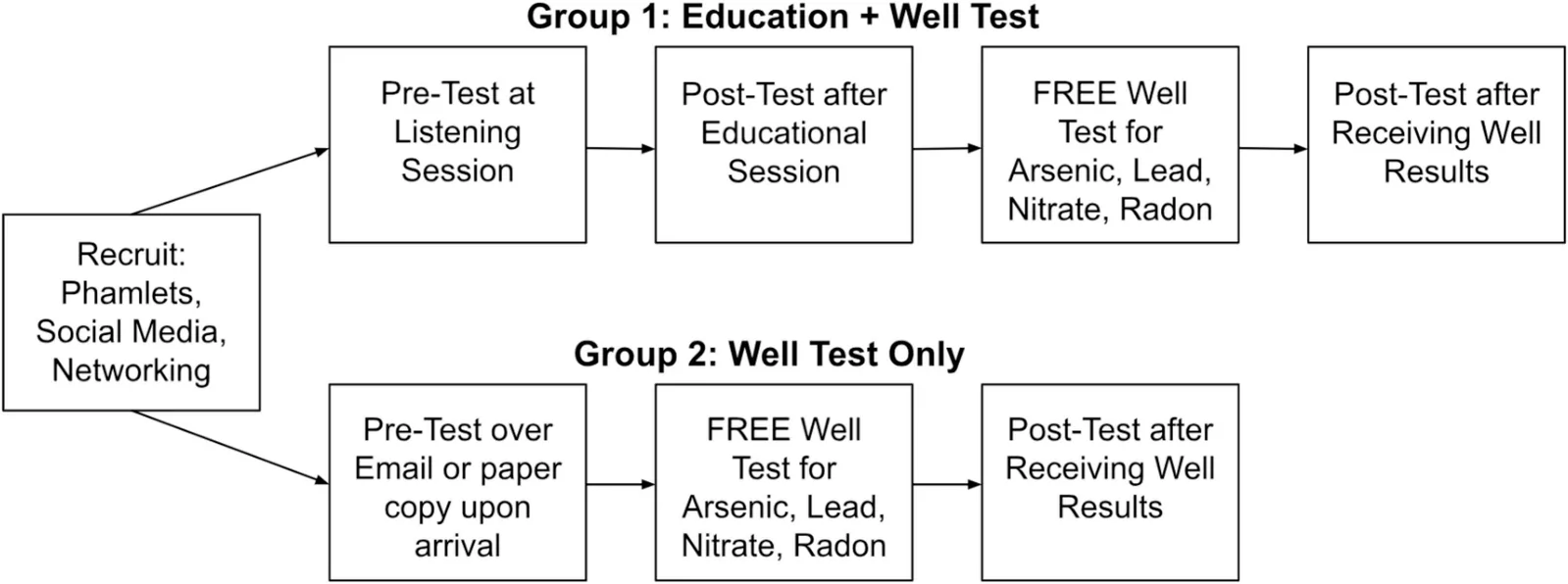
Study Methodology Among Rural Private Well Owners in Southeast Georgia, United States, July–October 2025.

Enrollment in Group 1 continued until the planned sample of 50 participants was reached. Subsequent eligible individuals who expressed interest in free private well testing, including individuals who did not participate in the church-based educational sessions, were enrolled in Group 2. Group 2 participants completed informed consent, eligibility screening, and the pre-intervention survey electronically before well testing was scheduled. They received free private well testing without the educational intervention and completed a post-testing survey after receiving their individual laboratory results. Enrollment continued until Group 2 reached 50 participants, resulting in a total sample of 100 private well owners. The Georgia Southern University Institutional Review Board approved the study protocol (IRB #H25302). The target sample size was determined by recruitment feasibility, available project funding, and laboratory testing capacity rather than an a priori power analysis. Study design and participant flow are illustrated in Fig. 1.

The educational intervention comprised five in-person, community-based listening sessions held during church dinners in Evans and Tattnall counties. The listening sessions used an interactive discussion format in which participants received information on regional cancer trends, cancer screening disparities, and potential health risks associated with private well contaminants, while also having opportunities to ask questions, discuss their experiences and concerns related to private well water, and provide feedback. Participants completed informed consent and the pre-intervention survey before the educational session and an immediate post-intervention survey following the session. Participants were subsequently contacted to schedule free private well water testing and received their individualized laboratory results by email.

### 2.3 Measures

Pre- and post-intervention surveys (SI1 and SI2) were administered through Qualtrics. The pre-survey assessed demographic characteristics, well management practices, cancer screening history, and CHBM constructs (perceived susceptibility, perceived seriousness, perceived benefits, perceived barriers, health motivation, and self-efficacy) using Likert-scale items adapted from the validated CHBM Scale and the National Cancer Institute's Health Information National Trends Survey (National Institutes of Health (NIH), 2022). The post-survey assessed changes in knowledge, understanding of well water results, and cancer screening intent. The primary outcome was cancer screening intent, measured as: “I intend to seek out cancer screening within the next year” (dichotomized: Yes/No). Cancer screening was intentionally assessed broadly rather than specifying a cancer type or screening modality, allowing participants to consider their individual risk factors, screening history and eligibility, and personalized well-water results when reporting screening intent. Internal consistency of CHBM constructs was evaluated using Cronbach's alpha; all constructs demonstrated acceptable reliability (SI3).

### 2.4 Statistical analysis

Descriptive statistics summarized participant characteristics and contaminant concentrations. Binary logistic regression models were conducted separately at baseline, following education (Group 1 only), and after receipt of well test results to evaluate predictors of cancer screening intent at each study phase. These models evaluated time-specific associations and did not account for within-person correlation across repeated measurements. Accordingly, comparisons of screening intent across study phases are presented as observed changes rather than estimates of longitudinal intervention effects. Predictor variables included CHBM constructs, demographic variables, and water quality findings. Adjusted odds ratios (AOR) and 95% confidence intervals (CI) were calculated. Analyses were conducted using IBM SPSS Statistics version 26; statistical significance was set at *p* < .05.

## 3 Results

**Fig. 2:**
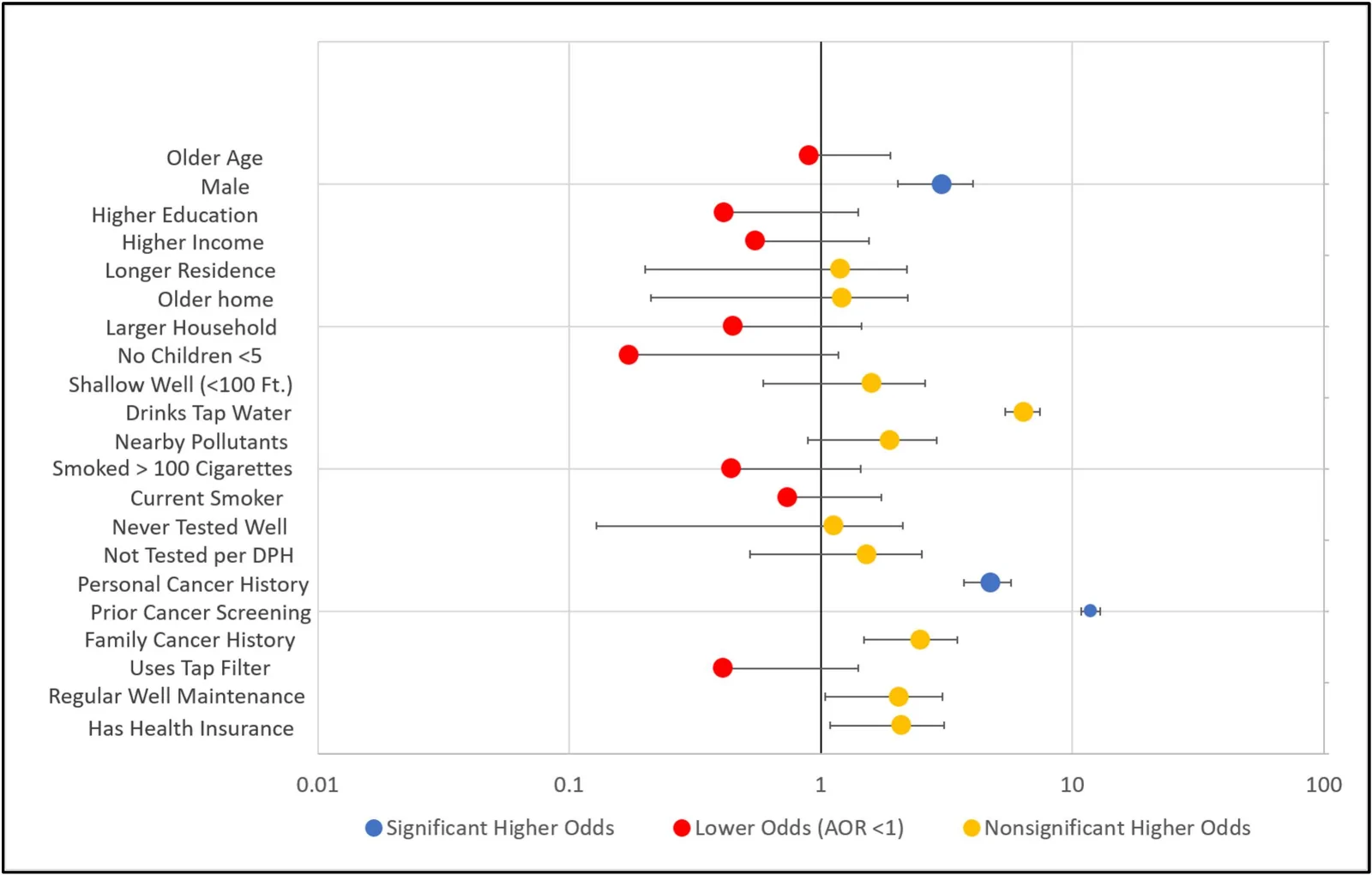
Baseline Factors Associated With Cancer Screening Intent Among Rural Private Well Owners in Southeast Georgia, United States, July–October 2025.

A total of 100 private well owners participated in the study. The sample comprised 52% females and 48% males; 97% identified as White. Most participants were aged 50 years or older (58%), and 48% reported a high school diploma as their highest educational attainment. Over half reported annual household incomes above $75,000. Most participants had health insurance (93%); however, only 44% had previously undergone cancer screening. Seventeen percent reported a personal cancer diagnosis, and 61% had an immediate family member with a cancer diagnosis (SI4). Male sex (AOR = 3.02, 95% CI: 1.16, 7.86) and personal cancer history (AOR = 4.71, 95% CI: 1.57, 13.12) were the strongest social predictors of screening intent at baseline. Fig. 2 displays the adjusted odds ratios and corresponding 95% confidence intervals for baseline factors associated with cancer screening intent, with estimates above and below an AOR of 1.0 indicating higher and lower odds of screening intent, respectively.

Private well testing revealed that 68% of sampled wells contained at least one contaminant. In 56% of wells, radon was detected between 300 and 4000 pCi/L, making it the most prevalent contaminant. Nitrate was detected in 22%, arsenic in 6%, and lead in 5% of samples. Contaminant prevalence was slightly higher in Group 1 (76%) than in Group 2 (60%).

Cancer screening intent increased substantially in both groups. In Group 1, intent rose from 30% at baseline to 62% following education sessions and remained at 62% after receipt of well results. In Group 2, intent increased from 20% at baseline to 56% after receipt of well test results. CHBM composite scores shifted across intervention phases: in Group 1, perceived susceptibility increased from a mean of 3.12 to 3.57, perceived benefits from 3.75 to 4.21, and self-efficacy from 3.75 to 4.76 between baseline and post-testing. Changes in Group 2 were comparatively modest; self-efficacy increased from 3.74 to 4.24, suggesting that individualized well results may strengthen behavioral confidence independent of formal education (Table 1, Table 2).

**Table 1:** Pre- and Post-Intervention Mean Scores Among Rural Private Well Owners in Southeast Georgia, United States, July–October 2025.

	Perceived Susceptibility	Perceived Seriousness	Perceived Benefits	Perceived Barriers	Health Motivation	Self- Efficacy
**Intervention Group 1**						
Pre-Test	3.12 (0.583)	3.23 (0.665)	3.75 (0.741)	2.75 (0.889)	4.03 (0.626)	3.75 (0.580)
Post-Education	3.48 (0.619)	3.54 (0.628)	4.04 (0.597)	2.64 (0.898)	4.38 (0.499)	4.18 (0.459)
Post Well Results	3.57 (0.863)	3.67 (0.801)	4.21 (0.642)	2.71 (0.909)	4.41 (0.445)	4.76 (0.316)
**Intervention Group 2**						
Pre-Test	3.01 (0.566)	3.29 (0.820)	3.79 (0.676)	2.45 (0.855)	4.05 (0.555)	3.74 (0.598)
Post Well Results	2.96 (0.826)	3.11 (0.832)	3.65 (0.846)	2.41 (0.943)	4.03 (0.613)	4.24 (0.618)

Note: Mean CHBM construct scores for Group 1 and Group 2 at baseline and post-interventions. Values represent mean scores with standard deviations (SD).

**Table 2:** Changes in Cancer Screening Intent by Intervention Group Among Rural Private Well Owners in Southeast Georgia, United States, July–October 2025.

Construct	Group	Intervention	AOR	95% CI	*p*-value
Perceived Susceptibility	1	Baseline	3.261	0.584–18.218	0.178
		Education	2.766	0.513–14.911	0.236
		Well Results	0.285	0.054–1.512	0.140
	2	Baseline	2.127	0.326–13.894	0.430
		Well Results	0.291	0.054–1.550	0.148
Perceived Seriousness	1	Baseline	0.111	0.016–0.787	0.028*
		Education	0.092	0.011–0.768	0.028*
		Well Results	2.346	0.456–12.065	0.308
	2	Baseline	0.484	0.108–2.165	0.342
		Well Results	0.847	0.144–4.990	0.855
Perceived Benefits	1	Baseline	14.402	1.094–189.521	0.043*
		Education	0.419	0.071–2.485	0.338
		Well Results	1.628	0.362–7.309	0.525
	2	Baseline	2.959	0.418–20.947	0.277
		Well Results	2.191	0.492–9.757	0.303
Perceived Barriers	1	Baseline	1.612	0.500–5.194	0.424
		Education	3.537	1.283–9.755	0.015*
		Well Results	1.392	0.619–3.131	0.424
	2	Baseline	0.823	0.261–2.597	0.740
		Well Results	0.791	0.349–1.789	0.573
Health Motivation	1	Baseline	0.259	0.020–3.424	0.305
		Education	0.414	0.043–4.025	0.448
		Well Results	0.878	0.120–6.415	0.898
	2	Baseline	2.566	0.192–34.278	0.476
		Well Results	1.346	0.246–7.377	0.732
Self-Efficacy	1	Baseline	0.752	0.145–3.890	0.734
		Education	9.142	1.025–81.512	0.047*
		Well Results	2.620	0.318–21.597	0.371
	2	Baseline	0.328	0.036–3.021	0.325
		Well Results	0.525	0.123–2.242	0.384

Note: Adjusted odds ratios (AOR) and 95% confidence intervals are shown for each CHBM construct. Models adjusted for demographic variables including age, sex, and prior cancer diagnosis. Adjusted odds ratios greater than 1 indicate increased likelihood of cancer screening intent. * indicates p < .05. ** indicates p < .01.

In logistic regression analyses, perceived benefits was the only CHBM construct significantly associated with screening intent at baseline in Group 1 (AOR = 14.40, 95% CI: 1.09, 189.52). Following the educational intervention, perceived barriers (AOR = 3.54, 95% CI: 1.28, 9.76) and self-efficacy (AOR = 9.14, 95% CI: 1.03, 81.51) emerged as significant predictors. Understanding of personalized well water results was the most robust predictor of screening intent when both groups were analyzed together (AOR = 4.75) (Table 3). Prior to the intervention, only one participant reported understanding their water quality status; after testing, 81 participants reported understanding their results. In Group 1, understanding results was associated with 19-fold greater odds of screening intent (AOR = 19.33, 95% CI: 2.18, 171.89); a similar magnitude was observed in Group 2.

**Table 3:** Predictors of Cancer Screening Intent Among Rural Private Well Owners in Southeast Georgia, United States, July–October 2025.

Construct	Group	Intervention	AOR	95% CI	*p*-value
Understand Contamination Risk	1	Education	3.750	1.056–13.311	0.041*
		Well Results	3.750	1.056–13.311	0.041*
	2	Well Results	2.538	0.735–8.770	0.141
Education Influence on Intent	1	Education	4.190	1.246–14.089	0.021*
Understanding Water Results	1	Well Results	19.333	2.175–171.886	0.008**
	2	Well Results	18.692	2.136–163.605	0.008**
	Both	Well Results	4.750	1.176–19.180	0.029*

Note. Adjusted odds ratios (AOR) and 95% confidence intervals are shown from binary logistic regression models predicting cancer screening intent. Models were adjusted for demographic covariates including age, sex, and prior cancer diagnosis. * indicates p < .05. ** indicates p < .01.

Detection of at least one contaminant was independently associated with increased screening intent across both groups (AOR = 2.67, 95% CI: 1.05, 6.77) (Table 4). Detection of radon specifically was associated with screening motivation (AOR = 2.67, 95% CI: 1.15, 6.21). Contaminant presence was a significant predictor in Group 2 (testing-only; AOR = 3.92, 95% CI: 1.13, 13.60) but not in Group 1. One possible explanation is that the educational intervention may have influenced participants' broader risk perceptions before receipt of their individualized well results; however, this potential mechanism was not directly tested. Studies using interaction or mediation analyses are needed to determine whether education modifies the relationship between contaminant detection and screening intent. Nitrate, arsenic, and lead were not significantly associated with screening intent, likely due to small detection counts limiting statistical power.

**Table 4:** Private Well Contaminant Detection and Odds of Cancer Screening Intent Among Rural Private Well Owners in Southeast Georgia, United States, July–October 2025.

Construct	Group	AOR	95% CI	*p*-value
Presence of any Contaminant	1	1.455	0.373–5.679	0.590
	2	3.923	1.132–13.602	0.031*
	Both	2.667	1.051–6.766	0.039*
Radon Present	1	1.625	0.507–5.213	0.414
	2	2.857	0.888–9.191	0.078
	Both	2.667	1.145–6.210	0.023*
Nitrate Present	1	1.500	0.423–5.315	0.530
	2	1.056	0.210–5.299	0.948
	Both	1.571	0.576–4.286	0.377
Arsenic Present	1	Not estimable	–	–
	2	Not estimable	–	–
	Both	Not estimable	–	–
Lead Present	1	2.111	0.204–21.873	0.531
	2	0.000	0.000–	1.000
	Both	2.786	0.300–25.884	0.368

Note. Adjusted odds ratios (AOR) and 95% confidence intervals are shown from binary logistic regression models predicting cancer screening intent. Models were adjusted for demographic covariates including age, sex, and prior cancer diagnosis. Arsenic models were not estimable due to sparse observations. * indicates p < .05. ** indicates p < .01.

## 4 Discussion

To our knowledge, this is the first study to apply the CHBM framework to environmental exposure communication as a pathway to cancer screening intent in a rural U.S. population. Our findings indicate that personalized private well testing was associated with increased screening intent and may have made environmental risk more personally meaningful, consistent with the CHBM's construct of cue to action. These results have important implications for health-equity-oriented cancer prevention practice and for integrating environmental health into public health policymaking.

The dramatic increase in participant understanding of water quality status from one participant at baseline to 81 after testing illustrates the power of personalized environmental feedback to activate risk awareness. Understanding of personalized well water results was the strongest predictor of screening intent in both groups, with effect sizes that exceeded those of most CHBM psychosocial constructs including perceived susceptibility, barriers, and health motivation. This pattern suggests that direct, individualized environmental feedback may be associated with screening intent beyond general health beliefs by making environmental risk more personally relevant and actionable. Dotherow et al. (2024) concluded that despite moderate awareness of water contamination risks among rural private well owners (M = 3.74/5), only 34% had ever tested their wells, illustrating that generalized knowledge is insufficient to motivate protective behavior. MacDonald Gibson et al. (2022) similarly found that generic risk information fell short, but that targeted, belief-informed communication increased engagement dramatically; participants who recalled receiving tailored risk messaging were more than 12 times as likely to report testing their water. Schultz et al. (2025) extended this evidence, demonstrating that community-tailored environmental health outreach increased both knowledge and actual testing behaviors within one year. Taken together, these findings suggest that individualized environmental feedback may reduce the psychological distance between environmental exposure and health motivation.

Both interventions increased screening intent, but the education-plus-testing approach produced greater shifts in CHBM constructs, particularly perceived barriers and self-efficacy. These findings are consistent with Health Belief Model literature demonstrating that multi-component interventions targeting perceived barriers, benefits, and self-efficacy produce the largest gains in cancer screening participation (Lau et al., 2020; Masoudiyekta et al., 2018; Rakhshani et al., 2024). Notably, the testing-only group showed meaningful self-efficacy gains and significant contaminant-driven increases in screening intent, suggesting that environmental information alone may be sufficient to motivate preventive behavior in the absence of formal health education. This has practical implications for resource-limited settings where education-intensive programs may not be feasible.

The association between radon detection and increased screening intent is noteworthy. Radon, a known lung carcinogen, is particularly underrecognized as a health threat in private well water (IARC, 2024; Grzywa-Celińska et al., 2020). Radon was the most prevalent contaminant detected in this study, with 56% of samples exceeding the 300 pCi/L action level used to interpret water results. However, radon detected in well water should be distinguished from established carcinogenic risk associated with radon exposure, particularly inhalation exposure. Thus, the observed association between radon detection and screening intent should not be interpreted as establishing a direct contaminant-specific cancer risk. Framing radon detection as a personally relevant cancer risk factor may activate CHBM constructs in ways that generalized public health messaging does not. Future interventions should connect specific contaminant findings to cancer risk and available screening options, providing actionable next steps proximal to the risk cue.

These findings contribute to growing evidence that social determinants of health, including unregulated water systems, rural geography, and limited access to preventive care, intersect to compound cancer inequities (O'Connor et al., 2018; Brock et al., 2024). Environmental risk communication that is personalized and embedded within preventive health frameworks may represent a promising, scalable strategy for addressing these compounding disadvantages. Partnerships between local health departments, Cooperative Extension programs, and community-based organizations could deliver integrated well testing and cancer prevention outreach at relatively low cost, particularly in communities with existing agricultural extension infrastructure.

Several limitations in this study should be noted. Participants were recruited from three rural Georgia counties, which limit generalizability to other populations of private well users. Although participants resided in rural counties characterized by community-level socioeconomic and healthcare access challenges, the enrolled sample was predominantly White (97%), most participants were insured (93%), and more than half reported annual household incomes above $75,000. Therefore, community-level rural and environmental disadvantage should not be interpreted as indicating individual-level socioeconomic disadvantage among all study participants. Future studies should include more racially and socioeconomically diverse populations of private well users.

Although participants completed surveys at multiple study phases, the logistic regression models were conducted separately at each time point and did not account for within-person correlation across repeated measurements. Therefore, observed changes in screening intent across study phases should not be interpreted as estimates of causal longitudinal effects. Future studies with larger, more diverse populations should employ longitudinal approaches that account for repeated observations within participants. The study cannot determine which specific cancer screening participants intended to pursue or whether that screening corresponded to current eligibility recommendations. This limitation also prevents contaminant-specific findings, such as the association with radon detection, from being linked to intent for a particular cancer screening modality. Recent clinical guidance has emphasized the importance of integrating private well education into preventive healthcare settings, particularly in rural and underserved communities where environmental exposures may go undetected for extended periods (Jegen et al., 2025). Future studies should evaluate environmental health education within healthcare settings as a strategy to increase exposure awareness and support preventive health behaviors.

## 5 Conclusion

Personalized environmental exposure information from private well testing was associated with increased cancer screening intent among rural private well owners in southeast Georgia. Understanding individualized well water results emerged as a robust predictor of screening motivation, operating independently of and in concert with formal health education. These findings support integrating environmental risk communication with cancer prevention strategies in rural, underserved communities. Programs linking free private well testing with clear, contaminant-specific health messaging may advance cancer prevention equity by activating the social-cognitive pathways the CHBM describes. Health policymakers should consider funding community-based environmental health outreach as a complement to existing cancer prevention infrastructure in rural areas.

## Studies in human

This study was performed in compliance with relevant laws, regulatory frameworks and guidelines where the research took place.

This study was approved by the The Georgia Southern University Institutional Review Board.

(Approval No. IRB #H25302)

## CRediT authorship contribution statement

**Victoria Clower:** Writing – original draft, Visualization, Methodology, Investigation, Formal analysis, Data curation, Conceptualization. **Andrew Hansen:** Writing – review & editing, Methodology, Conceptualization. **Jeff Jones:** Writing – review & editing, Methodology, Conceptualization. **Asli Aslan:** Writing – review & editing, Writing – original draft, Supervision, Resources, Project administration, Methodology, Investigation, Funding acquisition, Conceptualization.

## Informed consent and patient details

Written informed consent to take part in the study and to publish the article has been obtained from all participants or their legal representatives. The privacy rights of participants have been observed.

## Research ethics statement

This study was conducted in accordance with the ethical standards of Georgia Southern University and was approved by the Georgia Southern University Institutional Review Board (IRB). Participation was voluntary, and all participants provided informed consent prior to enrollment. Eligible participants were private well owners aged 18 years or older residing within study boundaries. Participants were informed of the study purpose, procedures, potential risks and benefits, and their right to withdraw from the study at any time without penalty. Data collection included survey responses and household private well water testing. Survey responses and laboratory results were linked using unique identification codes and were stored securely to protect participant confidentiality. All collected data were anonymous and confidential. All data were de-identified prior to analysis, and no personally identifiable information is reported in this manuscript. Participants who completed study activities were eligible for incentives as described in the informed consent process. All study procedures were conducted in accordance with applicable ethical guidelines for research involving human participants. Ethical approval was obtained from the lead authors' university institution (2025/H25302).

## Funding

This work was supported by the Evans County Cancer Association Relief Effort and Support (CARES) Foundation.

## Declaration of competing interest

The authors declare no competing financial interests or personal relationships that could have appeared to influence the work reported in this paper.

## Data Availability

Data will be made available on request.
